# Breakthrough *Magnusiomyces clavatus* bloodstream infection with silent dissemination, following allogeneic hematopoietic stem cell transplantation

**DOI:** 10.1016/j.mmcr.2026.100791

**Published:** 2026-05-06

**Authors:** Almahmoudi Nawal, Anthony Lieu

**Affiliations:** Division of Infectious Diseases, Department of Medicine, University of British Columbia, Canada

**Keywords:** *Geotrichum clavatus*, *Magnusiomyces clavatus*, *Saprochaeta clavate*, Breakthrough fungemia, Rare yeast infections

## Abstract

Magnusiomyces species have emerged as a cause of pulmonary and disseminated infections in immunocompromised patients, particularly those with hematologic malignancies and/or hematopoietic stem-cell transplantation. These organisms are associated with a high mortality rate, particularly when dissemination occurs. We report a case of *Magnusiomyces clavatus* fungemia with silent dissemination following allogeneic hematopoietic stem cell transplantation, involving the lungs and spleen. This highlights the importance of early recognition, microbiological identification and consideration of CT imaging to detect occult dissemination, which critical for guiding appropriate management.

## 1 Introduction

*Candida* and *Aspergillus* remain the most dominant causes of invasive fungal diseases (IFD) in patients with hematologic malignancies (HM) and hematopoietic stem cell transplant (HSCT). However, the widespread use of antifungal prophylaxis in HM and HSCT recipients, along with improved microbiological identification methods, has led to an increase in breakthrough infections caused by resistant or rare fungi [1,2]. Among these, *Magnusiomyces clavatus* is now recognized as an emerging cause of bloodstream infections in immunocompromised patients, particularly in the setting of prolonged neutropenia or corticosteroids used [3,4].

Clinical presentation can be subtle, and while imaging is not routinely indicated for transient fungemia unless clinical signs are present, we present a case of transient *Magnusiomyces clavatus* bloodstream infection with clinically silent pulmonary nodules and multiple splenic hypodense lesions, consistent with disseminated infection.

## 2 Case presentation

**Fig. 1:**
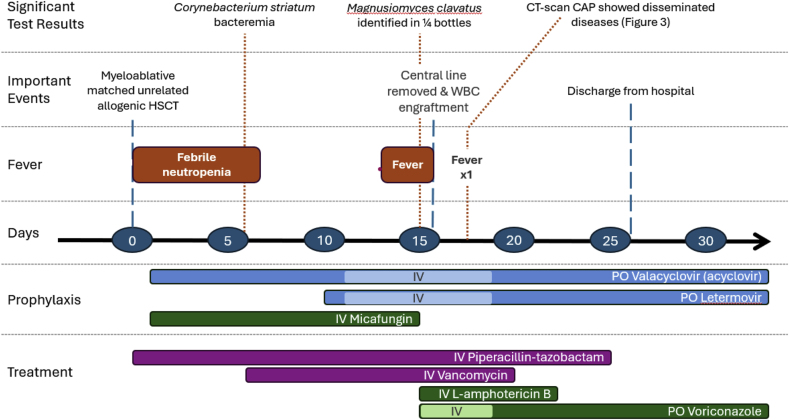
Clinical timeline of disseminated Magnusiomyces clavatus infection following allogeneic HSCT Footnote: abbreviation CAP, chest, abdomen and pelvis; HSCT, hematopoietic stem cell transplant; WBC, white blood cell; IV, intravenous; PO, per os; L-amphotericin B, liposomal-amphotericin B.

A 60-year-old male with a history of treated follicular lymphoma was admitted for an allogeneic HSCT due to therapy-related myelodysplastic syndrome. He underwent myeloablative conditioning with busulfan, fludarabine, and anti-thymocyte globulin for his matched unrelated donor HSCT (day 0 defined as his transplant date). He was also given graft-versus-host disease prophylaxis with cyclosporin and methotrexate. For antimicrobial prophylaxis, he received valacyclovir and micafungin on day +1, and letermovir on day +10 (Fig. 1).

His early post-transplant course was complicated by febrile neutropenia, which was treated with intravenous piperacillin-tazobactam. His chest computed tomography (CT) scan showed no significant abnormalities. On day +6, his blood cultures were positive for *Corynebacterium striatum,* consistent with a central line-associated bloodstream infection, as indicated by time-to-positivity analysis. Given his significant mucositis, which required patient-controlled analgesia pump and total parenteral nutrition, a central line salvage strategy was attempted. Intravenous vancomycin was added, and he subsequently defervesced and cleared his bacteremia within 48 hours.

On day +11, he developed recurrent fever while still on intravenous vancomycin and piperacillin-tazobactam. His nasopharyngeal swab, blood adenovirus PCR, and repeated blood cultures all returned negative. His fever persisted for an additional four days. On day +15, while still receiving micafungin prophylaxis, one of four blood culture bottles from the central line yielded a positive result for fungal elements. The blood culture bottle Gram stain revealed septate hyphae, and the isolate grew on Sabouraud dextrose agar as white, dry, and slightly cottony colonies (Fig. 2a, Fig. 2ba and b).

**Fig. 2a:**
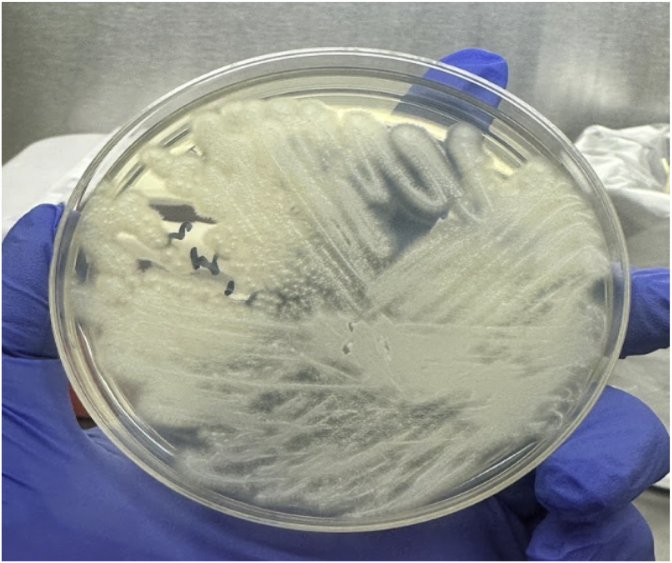
Blood culture on Sabouraud dextrose agar grew as white, dry and slightly cottony colonies after incubation at 28 ± 2 °C in ambient air.

**Fig. 2b:**
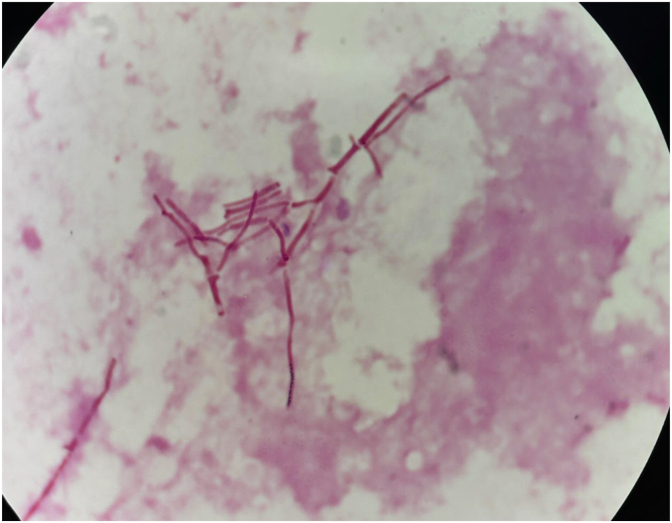
Direct microscopy of Blood Culture: Gram stain of the positive blood culture bottle reveals septate hyphae. Magnification 1000x.

At that time, he had started engrafting, and his absolute neutrophil count (ANC) had reached 0.8 × 10^9^/L. His tunnelled central venous line was promptly removed. Micafungin was discontinued, and intravenous voriconazole was initiated at increase dose (6mg/kg q12h), to account for the known interaction resulting in reduction in voriconazole with letermovir, along with liposomal amphotericin B (5mg/kg daily) pending final identification of fungi. Intravenous administration of voriconazole was given due to severe mucositis. Subsequent peripheral blood cultures remained negative. Identification was obtained using matrix-assisted laser desorption ionization-time of flight mass spectrometry (MALDI-TOF MS) (Bruker Biotyper), with confirmation using the MSI database version 2.0 (score 26.48, category A). The isolate was identified as *Magnusiomyces clavatus*, formerly known as Saprochaete clavate, and *Geotrichum clavatum*. Given the lack of established breakpoints for this organism and as his blood cultures rapidly cleared, minimum inhibitory concentration for antifungal was not performed by the medical microbiology laboratory.

While on combination antifungal therapy and despite having solid counts, his fever recurred. Repeat CT imaging of the chest, abdomen, and pelvis on day +17 showed innumerable new bilateral pulmonary nodules and multiple hypodense splenic lesions, consistent with disseminated fungal infection (Fig. 3a). His fever eventually resolved on antifungal. Following identification as *Magnusiomyces clavatus*, liposomal amphotericin B dose was reduced to 3mg/kg. In addition, once voriconazole therapeutic levels was achieved, amphotericin was discontinued after 7 days of combination therapy. Voriconazole was subsequently transitioned to oral therapy once mucositis improved and oral intake was tolerated.

**Fig. 3a:**
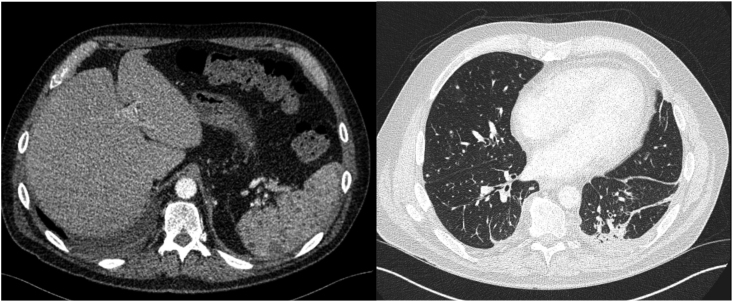
CT scan of the chest and abdomen done at Day +22 (3 weeks and 1 day) showing innumerable new bilateral pulmonary nodules and splenic lesions, suggestive of disseminated fungal infection.

Follow-up imaging at 4 weeks demonstrated near-complete resolution of splenic lesions and marked improvement in pulmonary nodules (Fig. 3b). He completed 10 weeks of voriconazole. Final CT imaging revealed complete resolution of the lung lesions (Fig. 3c).

**Fig. 3b:**
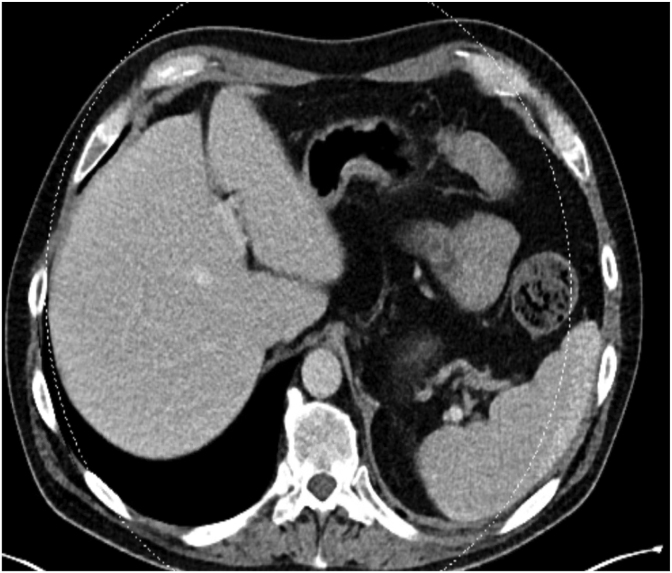
Follow up CT scan of the abdomen at Day+48 (6 weeks and 6 days) showing complete resolution of the previously splenic abscess.

**Fig. 3C:**
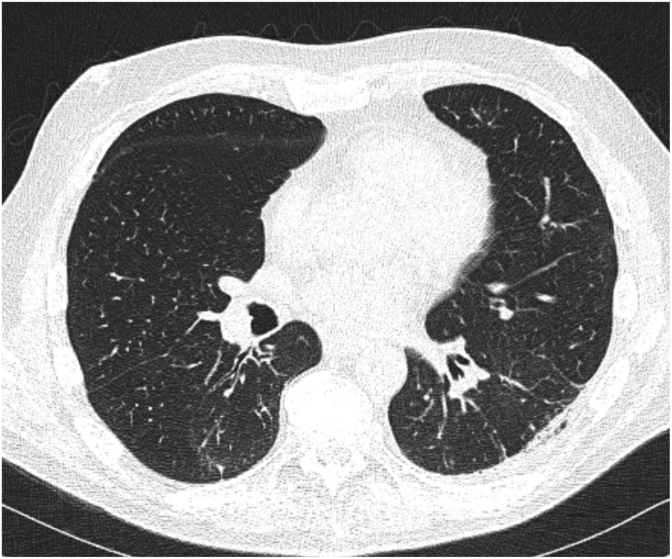
Follow up CT scan of the abdomen at Day+77 (11 weeks) showing complete resolution of previously bilateral pulmonary nodules.

## 3 Discussion

We present a case of disseminated infection caused by *Magnusiomyces clavatus* in a patient undergoing allo-HSCT for myelodysplastic syndrome, who was receiving micafungin prophylaxis. Historically, fungal taxonomy relied primarily on morphological characteristics. The genus *Geotrichum* is defined as monomorphic, yeast-like fungus that produces characteristic non-alternating, rectangular arthroconidia. With the advent of molecular techniques, phylogenetic analysis of rDNA sequencing and later multilocus sequence typing revealed significant phylogenetic divergence within the genus *Geotrichum*. This has led to its taxonomic revision into multiple genera, including *Saprochaeta* and *Magnusiomyces.* For instance, *Geotrichum clavatum* was initially reclassified as *Saprochaeta clavata* and later as *Magnusiomyces clavatus* based on multilocus phylogenetic data [1]. *Magnusiomyces* spp. are opportunistic fungi found in the environment, including soil, water, wood, and dairy products, household dishwashers, and occasionally colonize the human microbiome [3,5,6]. *M. clavatus* has been described in bloodstream infections, particularly in individuals with HM or following HSCT [3]. Neutropenia is the most significant risk factor, with other contributing factors including disruption of mucosal barriers, the use of central venous catheter, and alteration of endogenous microbiota due to the use of broad-spectrum antibiotics [7].

In this case, there were two possible sources: the organism was isolated from the central venous culture, raising the possibility of a catheter-associated infection. In contrast, given the presence of severe mucositis, gastrointestinal translocation remains another plausible source of infection.

A breakthrough infection with *Magnusiomyces* spp. including *M. clavatus* has been reported mostly with prophylactic echinocandins and posaconazole, raising concerns for intrinsic or acquired resistance mechanisms [1]. The absence of established breakpoints or epidemiological cut-off values (ECV) for *M. clavatus* complicates therapeutic decision-making. Current guidelines recommend against the use of echinocandins due to high MIC distribution and potential mutations in the FKs1P HS1 region, which may confer resistance [8,9]. Fluconazole also demonstrates limited in vitro activity, with elevated MIC distribution, and posaconazole MIC_90_ values have been reported at 1 to 2 mg/L, which may indicate suboptimal efficacy [10]. The 2014 ESCMID and ECMM joint guidelines for management rely on retrospective case series and expert opinion, recommending the use of liposomal amphotericin B, with or without voriconazole or flucytosine [4,11]. These recommendations are supported by reported MIC_90_ values of <1 mg/L for amphotericin B, itraconazole, voriconazole, and flucytosine [10].

While antifungal therapy is essential for the management of *Magnusiomyces spp.* bloodstream infections, the primary factor linked to survival was neutrophil recovery [1]. A consistent feature across reported *M. clavatus* bloodstream infections is the high rate of dissemination, often involving the spleen, liver, and lungs. In our case, radiographic evidence of dissemination was identified despite transient fungemia, early antifungal therapy, and prompt removal of the central venous catheter. Similarly, in an Italian cluster, three patients developed asymptomatic splenic involvement following a brief febrile episode, with dissemination detected on imaging before blood culture became positive, highlighting its ability for rapid and clinically silent stealth dissemination in neutropenic hosts [12].

Among 75 published cases over the last 10 years in the literature, 48 cases (64.0%) exhibited evidence of dissemination, including 31 cases (41.3%) with pulmonary involvement and 30 cases (30/53, 56.6%) with gastrointestinal involvement, which were predominantly liver or splenic involvement (Table 1). These findings of high proportion of dissemination emphasize the importance of obtaining a CT chest, abdomen, and pelvis in febrile neutropenic patient with *M. clavatus* fungemia, as signs and symptoms alone may underestimate disease extent and delay therapeutic decisions or source control.

**Table 1:** Reported cases of *Magnusiomyces clavatus* bloodstream infection (2015-2025) with clinical details and proportion of disseminated diseases.

Reference	Number	Hematologic malignancy	Echino-candin prophylaxis	Triazole prophylaxis	Dissemi-nated disease	Pulmonary Involvement	Gastro-intestinal involvement
Del Principe et al. (2016) [12]	3	3/3	0/3	1/3	3/3	2/3	3/3
Favre et al. (2016) [13]	1	1/1	1/1	0/1	1/1	1/1	1/1
Buchta et al. (2019) [7]	6	6/6	0/6	1/6	4/6	1/6	3/6
Stanzani et al. (2019) [14]	3	3/3	3/3	0/6	3/3	2/3	2/3
Leoni et al. (2019) [15]	1	0/1	0/1	0/1	1/1	1/1	1/1
Liu et al. (2019) [11]	1	1/1	1/1	0/1	1/1	1/1	1/1
Noster J et al. (2022) [4]	24	20/24	NA	NA	19/24	10/24	12/24
Pasqualone et al. (2022) [16]	1	1/1	0/1	0/1	1/1	1/1	1/1
Del Principe et al. (2023) [1]	30	30/30	7/30	5/30	10/30	9/30	NA
Mitchel et al. (2023) [17]	1	1/1	1/1	0/1	1/1	1/1	1/1
Alzahrani et al. (2025) [18]	1	1/1	0/1	1/1	1/1	1/1	NA
Sarika et al. (2025) [19]	3	3/3	3/3	0/3	3/3	1/3	2/3
Total (%)	75	70/75 (93.3)	16/51 (31.4)	11/54 (20.4)	48/75 (0.64)	31/75 (41.3)	30/53 (56.6)

*Magnusiomyces clavatus* is an uncommon but highly pathogenic fungus in immunocompromised patients, particularly those with HM or recipient of allogeneic HSCT undergoing induction or myeloablative chemotherapy. Our case highlights the importance of recognizing these pathogens as a possible breakthrough fungal infection despite echinocandin or posaconazole prophylaxis and obtaining early CT imaging of the chest, abdomen, and pelvis to detect occult dissemination. In the absence of prospective trial data, early recognition, initiation of adequate antifungal therapy, source control, and measures to expedite immune reconstitution are key to improving the traditionally poor outcomes associated with this rare yeast.

## CRediT authorship contribution statement

**Almahmoudi Nawal:** Writing – review & editing, Writing – original draft, Project administration. **Anthony Lieu:** Writing – review & editing, Supervision.

## Conflict of interest

The authors have no competing interests to declare.
